# Biological Basis for Cerebral Dysfunction in Schizophrenia in Contrast with Alzheimer’s Disease

**DOI:** 10.3389/fpsyt.2013.00119

**Published:** 2014-02-03

**Authors:** Rodrigo O. Kuljiš, Luis V. Colom, Leonel E. Rojo

**Affiliations:** ^1^Brain-Mind Project, Inc., Galveston, TX, USA; ^2^Institute of Ethnopharmacology, Universidad Arturo Prat, Iquique, Chile; ^3^The University of Texas Medical Branch at Galveston, Galveston, TX, USA; ^4^Instituto Neurogeriátrico, Santiago, Chile; ^5^Zdrav Mozak Limitada, Santiago, Chile; ^6^Clínica Las Condes, University of Chile, Santiago, Chile; ^7^Encephalogistics, Inc., Miami, FL, USA; ^8^The University of Texas at Brownsville, Brownsville, TX, USA

**Keywords:** Alzheimer’s disease, schizophrenia, cognition, behavior, dementia, *dementia praecox*

## Abstract

Schizophrenia and Alzheimer’s disease are two disorders that, while conceptualized as pathophysiologically and clinically distinct, cause substantial cognitive and behavioral impairment worldwide, and target apparently similar – or nearby – circuitry in regions such as the temporal and frontal lobes. We review the salient differences and similarities from selected historical, nosological, and putative mechanistic viewpoints, as a means to help both clinicians and researchers gain a better insight into these intriguing disorders, for which over a century of research and decades of translational development was needed to begin yielding treatments that are objectively effective, but still very far from entirely satisfactory. Ongoing comparison and “cross-pollination” among these approaches to disorders that produce similar deficits is likely to continue improving both our insight into the mechanisms at play, and the development of biotechnological approaches to tackle both conditions – and related disorders – more rapidly and efficaciously.

## Introduction

Cognitive and behavioral dysfunction occurs both in Alzheimer’s disease (AD) and in schizophrenia, and – differences aside – is one of the most distinctive and disabling features of these otherwise conceptually different disorders. Therefore, an assessment of the differences and similarities in the cognitive impairment in schizophrenia vs. AD is relevant for many reasons, including: (1) both disorders are among the most prevalent neuropsychiatric disorders worldwide, (2) the term *démence précoce* coined by the French alienist Auguste Morel in 1853, which was then translated as *dementia praecox*, by Pick in Prague (1) and then popularized by Eugen Bleuler (2) in 1911 and, especially, by Emil Kraepelin (3) still conjures a misleading association with the degenerative dementias (4). In fact, although the term *dementia praecox* has since fallen out of favor, it remains in the memory of both specialists and, in many cases, the public, and still evokes valid parallels as well as easily understandable confusion and erroneous comparisons with and array of conditions that we now conceptualize as nosologically distinct, i.e., the distinct types of Degenerative Dementias, and (3) the increasingly better documented tendency of most patients with schizophrenia to develop cognitive dysfunction and imaging evidence for brain degeneration over the years, even if they go into remission and/or have a seemingly favorable response to treatment. This constitutes another important feature in common with the dementias due to cerebral degeneration, since the clinical manifestations of patients that have suffered from schizophrenia eventually tend to blend and merge with the array of symptoms in the dementias more prevalent in the elderly. Since the latter phenomenon casts yet another layer of controversy about the already confusing – and conflicting – hypotheses about nosology in schizophrenia, it is both appropriate and necessary to discuss this matter in sufficient detail to inform the efforts to help develop innovative and transformative biotechnology to conquer these and related disorders.

## Historical Overview

The controversy over the appropriateness of the term *dementia praecox* usually does not revolve around its attempt to capture or subsume the rather vague concepts of “premature dementia” and “precocious madness.” In that sense, many would agree that Morel’s coinage does convey adequately the concept of a premature dissolution of cognitive function, or at least an impairment of the ability for cognitive integration. AD was characterized from the start as a disorder of cognitive integration, with prominent psychiatric features (5) and soon after its discovery it was postulated that it represented a pre-senile condition (6). That latter feature – abandoned only very gradually since the late 1970s – attempted to position AD between the “premature” dementias (e.g., schizophrenia), and to justify the now nearly vanished distinction with the dementias in “senility.” This is both because the concept of senility has become increasingly more elusive biologically, and because clinical and histopathological differences could not be substantiated comparing patients in their fourth through seventh decades (5), although that may change as we become able to evaluate large numbers of much older patients (7). Be that as it may, it does not call into question the concept that schizophrenia usually presents earlier in life than AD, including most of the extremely rare cases of early-onset so-called familial AD (early-to-mid third decade). Historically, there was never a confusion about the use of the word “dementia” in the sense of erroneously implying a strict similarity with dementias of later onset, whether “pre-senile” or “senile.” In fact, Bleuler never implied this, although he did advance the concept that schizophrenia is a disorder from which no one recovers (2), which was refuted by Kraepelin much later. Nevertheless, it was Kraepelin, employing his careful analysis of large numbers of patients, first in Dorpat and then in Heidelberg, that formulated the notion that schizophrenia was primarily a “psychic degenerative process” (1899) within which he distinguished several different subclasses, including “*dementia simplex*” (roughly today’s simple schizophrenia) (3). It is beyond the scope of this chapter to discuss in depth the fact that, despite an agreement regarding the primacy of the cognitive manifestations in schizophrenia, there are many important differences between Bleuler’s and Kraepelin’s formulation of the concept of *dementia praecox*. The key fact remains, however, that early formulations of the concept of schizophrenia emphasized a notion of cognitive deterioration and relatively relentless progressive deterioration, a concept that was eventually restored after some six or more decades of predominance of psychoanalytic views that painted – unfortunately inaccurately – a less dire prognosis over time.

## Comparison between the Clinical Manifestations in Schizophrenia and in AD

Alzheimer’s disease usually begins with a progressive deterioration in memory, and over time additional symptoms accrue that reflect the involvement of other cognitive spheres. The latter symptoms may include aphasia, apraxia, agraphia, and alterations in visuospatial abilities (6, 8) and eventually a disintegration of virtually every cognitive sphere. There are also a few well-documented cases in which – uncharacteristically – instead of memory loss, the initial manifestation involves a different cerebral cortex-dependent faculty, such as an aphasia, “posterior cortical atrophy,” or a right parietal syndrome (9–17).

The bulk of the experimental evidence suggests very strongly that this is a predominantly cerebrocortical disorder, first recognized by the prescient statement of Alois Alzheimer himself (5) that this is a disorder of the cerebral cortex. Within the general framework of cortical involvement, however, there is a clear predilection not only for certain regions of the cortex, but also for certain layers of this structure and, as is well known, for certain types of neurons: pyramidal cells (18). Here, after reviewing briefly the latter aspects, we end this section by focusing on a remarkable but essentially ignored topic: the mounting evidence that there is also a predilection for specific components of the hypothetical anatomical and functional unit of the cerebral cortex, the so-called cortical “module.” This relatively new pathophysiological concept has key implications for the understanding of the disorder from the integrative and systems neuroscience perspective (19). Such viewpoint may permit a new synthesis with the inevitably – and in fact, desirably – reductionist analysis of the disorder from the molecular perspective, since it allows for an integration of these two distinct levels of inquiry in order to better understand the basis for normal and disordered cognitive function and behavior in virtually all neuropsychiatric conditions (20, 21). Along these lines, it would seem that – given the volume of information available – this novel paradigmatic view may eventually be applied to the understanding and treatment of many other disorders characterized by deterioration in cognition and behavior due to targeting of the cerebral cortex, such as schizophrenia.

## Alzheimer’s Disease as a Cerebrocortical Process

Despite the broad variety of symptoms among patients – including unusual presentations and even those that are extremely rare such as aphasias and a right parietal lobe syndrome – it seems reasonable to postulate that in virtually all cases the symptoms reflect cerebrocortical dysfunction (5, 18–24). The vast majority of studies also indicate that the main lesions situated in the brain – i.e., senile plaques and neurofibrillary tangles – are situated predominantly in the cerebral cortex (5, 25). Furthermore, even in those studies focusing on the subcortical involvement, such as those in the nucleus basalis of Meynert (26), in the *locus ceruleus* (27) or in the pulvinar nucleus of the thalamus (18), the pathophysiological interpretation of the findings is invariably made in terms of how they affect cortical function, and in no case contradict the fundamental importance attributed to the cortical lesions. Thus, it is postulated that lesions in the nucleus basalis of Meynert are important because they deprive the cerebral cortex of its cholinergic innervation (25), and that lesions in the pulvinar destroy cortico-thalamo-cortical circuits that interconnect this nucleus with a vast territory in the association cortex, thus compounding the functional impact of cortico-cortical disconnection in AD (18). There is virtually no information as to whether such connections are targeted in schizophrenia, which is surprising since such a phenomenon – whether due to anatomical damage or “functional” mechanisms (e.g., synaptic failure without synaptic degeneration) – would likely result in a dissolution of the integration of cortical functions that is presumably essential for normal cognition and behavior.

**Figure 1:**
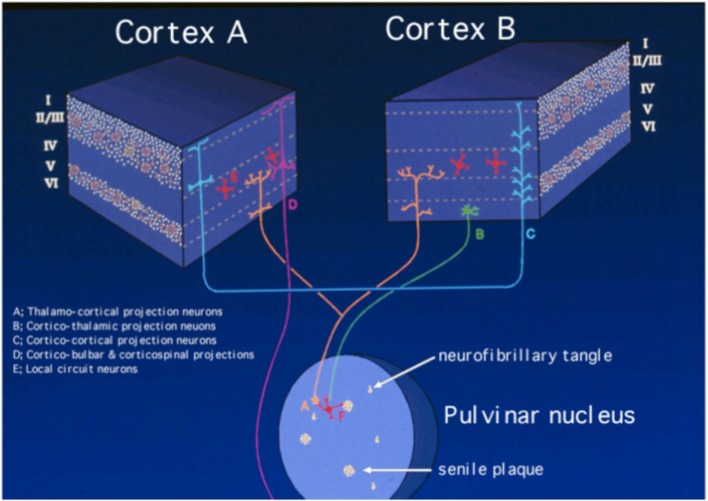
**Schematic representation of the neuropathology of Alzheimer’s disease, based on the selective laminar distribution of senile plaques and neurofibrillary tangles and the circuitry they target**. The blocks represent different regions of the cerebral cortex, in which the Roman numerals indicate cortical layers and letters identify main types of neurons according to the key in the figure itself. Neurons selectively targeted in AD are the medium-sized pyramidal neurons making cortico-cortical connections (labeled as “C”) and the corticipetal neurons in the pulvinar nucleus of the thalamus that effect cortico-thalamo-cortical connections (labeled as “A”). By contrast, large pyramidal neurons in layer V (labeled as “D”), corticothalamic projection neurons in layer VI (labeled as “B”), and layer IV granule cells (labeled as “E”) are much less affected or spared. The putative involvement of local circuit neurons (labeled as “F”) is less well understood. Modified from Kuljiš (21).

These notions, combined with the relative absence of lesions in the majority of subcortical regions – and especially in contrast with many other dementing conditions in which the pathology is situated predominantly (if not always exclusively) subcortically (e.g., Parkinson’s disease dementia, Huntington’s disease, and Progressive Supranuclear Palsy) – support the contention that AD is a cerebrocortical disconnection disorder (18, 28–32). This concept has allowed some of us to focus many studies in which we attempt to understand in detail the pathophysiology of the disorder, by analyzing in detail the topographic distribution of the lesions in the cerebral cortex (23, 27, 33, 34). Such focus intends, among other objectives, to understand the mechanisms underlying the disorder in terms of the circuits that are presumably selectively affected, compared with circuits that tend to be relatively spared, or are resistant to the degenerative process. Such a perspective is rarely considered in parallel with that which tends to dominate inquiry in this field today: i.e., the molecular factors involved in the pathogenesis and pathophysiology of the disease. Nevertheless, the analysis of the patterns of topographic distribution of lesions in the disorder, which is in its infancy, adds an important dimension – i.e., that of Systems Neuroscience – to the understanding of the pathophysiology of the condition. Such a perspective is undoubtedly essential in the brain, which is the organ with the broadest phenotypic diversity among its cells in the entire organism, a diversity that cannot be subsumed, or “reduced” to the molecular level alone. Therefore, it is not reasonable to consider the role of any trophic or toxic agent – or any other molecular factor affecting the expression of the clinical phenotype – while ignoring the highly complex three-dimensional organization of the brain. In fact, assuming that most of the various cell types in the brain are exposed to the degenerative process, the bulk of the neuropathological studies demonstrate that specific types of cells are highly susceptible, whereas others are remarkably resistant to the disease (Figure 1). Therefore, the diversity of the potential targets contrasts sharply with the relatively small array of apparently affected cells, a fact that defies explanation to this date.

**Figure 2:**
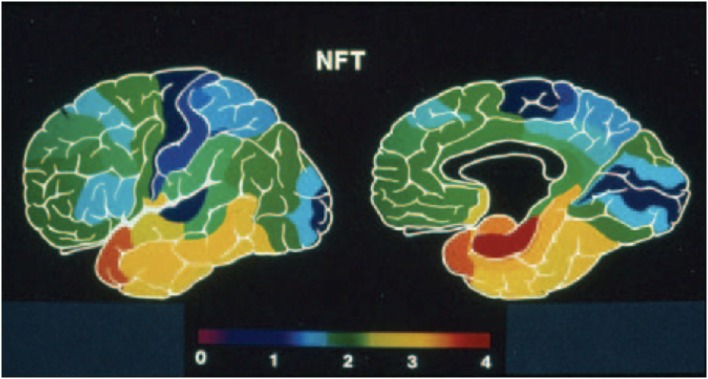
**Pseudocolor rendering of the relative density of neurofibrillary tangles (NFT) in Alzheimer’s disease, where warm colors represent higher densities and cold colors progressively lower lesion densities**. Modified from the work of Arnold and collaborators (22), and from Kuljiš (33).

The great majority of studies on AD reveal a considerably stereotyped pattern of involvement in the cortex: regions most affected include the entorhinal cortex (Brodmann’s area 28), the perirhinal cortex (area 35), the subjacent hippocampus and the temporopolar cortex (area 38) (22–24, 34). Compared to these areas, the rest of the association cortex develops an intermediate density of lesions, whereas the primary cortices – both motor and sensory – develop the lowest density of neurofibrillary tangles (Figure 2). It is important to emphasize, however, that this notion is predicated primarily on the density of neurofibrillary tangles, whose distribution is felt by many to correlate best with the level of cognitive deterioration (35–37), although it has been shown that primary sensory cortices have a high density of senile plaques (38) which is in fact much higher than that in medial temporal regions that are widely perceived as severely affected (22). Apart from this gross overall topographic pattern, there is also a distinct laminar predilection in the distribution of the lesions (Figure 1). This pattern applies both to allocortical and to iso(neo)cortical regions, and consists generally in a higher density of lesions in layers from which cortico-cortical projections originate (layers II/III in the neocortex), as compared with layers that receive projections from subcortical regions (I and IV) or layers from where feedback cortico-subcortical projections originate (VI). Such a pattern permits postulating that the disposition of the lesions explain the cortical dysfunction in terms of a disconnection resulting from the disruption of cortico-cortical connectivity (32, 38), as already discussed above and summarized in Figure 1. In addition to such regional and laminar patters of selective targeting, there is mounting evidence that there is also selective targeting of supracellular arrays that constitute the hypothetical functional units of the cerebral cortex, often called “modules” or “hypercolumns,” which we have reviewed in detail recently (19). Therefore, as befits the complexity of the cerebral cortex, there is an intricate pattern of selective vulnerability vs. sparing of closely intertwined cells, and groups of cells, that is only beginning to become apparent, but that needs to be better understood to achieve an adequate understanding of the mechanisms governing the targeting of molecules, cells, cellular arrays, and cerebrocortical regions in AD.

## Schizophrenia as “The Graveyard of Neuropathologists”

Many believe that schizophrenia and related disorders result from alterations in brain circuits (39). Yet, this probably correct assumption lacks a concrete factual demonstration – hundreds of unverified neuropathological claims non-withstanding – and is in stark contrast with the relative wealth of information on the neuropathology of AD because virtually nothing claimed about histopathological observations in schizophrenia has survived efforts at independent verification or corroboration. This justifies Stevens’ admonition about schizophrenia being “the graveyard of neuropathologists” (40, 41), which unfortunately continues to be the case decades since its enunciation. In fact, arguably the only widely accepted, consistent morphological alterations have been found by *in vivo* imaging, that consist in a reduction in gray matter, enlargement of the ventricles of the brain, and focal alterations in the white matter (42–44). This is hardly specific, much less pathognomonic, and is perhaps consistent with the increasingly popular syndromatic – as opposed to a unique, or “sole disease” – view of the disorder (45), which would be consistent with the lack of a truly unique and unanimously agreeable cellular and molecular neuropathology. Be that as it may, it justifies the still vaguely articulated perception that schizophrenia is a disease of the cerebral cortex (39).

To many, this might appear as a profoundly unsatisfying explanation, because it may imply that it is impossible to “pin down” schizophrenia to a specific disorder of one cell, one molecule, or one brain circuit, among other possibilities that may seem better suited to present conventions to develop a therapeutic armamentarium of modern Neuroscience. However, while the identification of a unique and consistent defect at any level – circuit, neural cell, or molecule – might facilitate enormously the rational design and development of treatments for any disease, nothing precludes the development and testing of novel agents for clinical syndromes – as opposed to diseases – which may be the best approach for schizophrenia. However, whether the condition is a syndrome due to many individually variable “factors” or “causes,” or a disease due to one and only one cause has profound implications for the design of rational strategies to pursue innovative treatments by means of drugs or other avenues.

The dopamine hypothesis’ origin provides an example of just such a situation, since it was formulated after the empirical observation that chlorpromazine improves the condition(s), and well before a rationale could be put together for the therapeutic effect based on what remained – and still remains – to be learned about that neurotransmitter and its roles in health and disease (46). The possibility that the seemingly logical focus thereafter on monoamine pathway-modifying pharmacological agents might have been inaccurate – from the pathophysiological perspective – does not negate the validity of searching for agents that maximize the benefits of chlorpromazine, while mitigating the potential for side effects. In fact, there are many lines of evidence that make it obvious that there’s more than a dopaminergic mechanism at play in schizophrenia, such as indications that there are also serotoninergic and glutamatergic defects involved as well (47–49), and there is also mounting evidence that there may be developmental alterations – which are not confined to the monoaminergic system – that either increase the risk or may even cause schizophrenia (50–53). In the latter (i.e., “developmental”) formulation, the clinical syndrome would be merely the symptomatic manifestation of a mild developmental disorder that manifests in the last phase of the brain’s development, i.e., as individuals undergo the transition from adolescence to adulthood, yet the fact that the clinical syndrome can occur well after (45), or even before (50) the adolescence-to-adulthood transition underscores the near certainty that this is not simply a sole disorder of brain maturation – however broadly one may one to define it – but a clinical syndrome that can occur as the result of many factors/causes, and not only at the very adolescence-to-adulthood interface.

Be that as it may, for the purposes of the present discussion, suffice it to say that there is a consensus that, like AD, schizophrenia targets mainly the cerebral cortex (Table 1) (39, 44, 45, 51). Accordingly, as mentioned at the very beginning, this warrants not only comparing these disorders both in terms of similarities and differences, but also in terms of the nosological dilemmas they present. The latter is not merely an “academic” exercise – in the often pejorative sense that this term is nearly synonymous with “useless” – but, on the contrary, has profound implications for how we conceptualize these diseases etiologically and pathophysiologically, which has extremely tangible and materially impactful consequences, among other things in the choice of biotechnological development investments we make to conquer them. In that latter sense alone, the difference amounts to millions every year, of whatever national currency is considered in terms of expenditures in health care.

**Table 1:** **A simplified list of selected similarities and key differences between Alzheimer’s disease and schizophrenia**.

	Alzheimer’s disease	Schizophrenia
Etiology	Unknown	Unknown
Brain regions targeted	Cerebral cortex including hippocampus, basal forebrain, pulvinar nucleus of the thalamus	Prefrontal cortex, hippocampus, and temporal neocortex
Cognitive impairment	Memory usually followed by additional cognitive and behavioral spheres of functioning	Impaired cognitive and behavioral integration, not usually preceded by isolated memory impairment
Average age at onset	Sixth decade and beyond	Early adulthood
Main neurotransmitters implicated	Acetylcholine	Dopamine, serotonin, glutamate
Reduced lifespan	Yes	Yes
Tendency for substance abuse	No	Yes
Additional comorbidities	Depression, anxiety	Depression, anxiety
Hallucinations, delusions	Yes	Yes
Social withdrawal, poor hygiene, motivation, and judgment	Yes	Yes
Catatonia	Very infrequent	Rarely present (subtype)
Social withdrawal, irritability, dysphoria	Yes	Yes
Psychosis	More likely in advanced stages	More likely in early stages
Remission possible	No	Yes
Developmental basis proposed	No	Suspected
Accepted evidence for cerebral degeneration	Precedes clinical manifestations	Occurs years or decades after symptom onset
Association with substance abuse/misuse	No	Yes
First-line treatment	Cholinesterase inhibitors, memantine	Antipsychotics
Accepted histopathological features	Yes	No
Biomarker(s) available	No^a^	No

*^a^Although literally dozens are proposed, but none accepted for clinical diagnosis*.

## Caveats on the Susceptibility of Comparisons between Schizophrenia and AD Due to Nosological Dilemmas

The “sporadic” (i.e., non-familial) form of the AD syndrome (sADs, where the first “s” is for “sporadic,” and the second “s” is for “syndrome”) is by far the most common form of this disorder, and appears to result from a more or less individually unique set of converging risk factors, inadequately counterbalanced by a set of hypothetical protective factors that is also relatively unique to the individual affected (20, 21, 54, 55). We have recently postulated (56) that these sets of opposing factors operate over decades – primarily (i.e., pathogenically) or secondarily (i.e., as a “common final pathway” that requires prior events) – to target the so-called “polydendrocytes,” a.k.a. beta astrocytes and the “fourth neuroglial type,” a relatively recently discovered (57, 58) and subsequently confirmed type of neural cell (59–62). We have re-designated them as the “Fourth Element” cell (4EC) following the tradition established by Santiago Ramón y Cajal when he made a distinction between neurons, macroglia, and all other types of cells in the brain, and lumped the latter types of cell into a “Third Element” (63). Pío del Río Hortega subsequently discovered – and named – what we know today as the microglia from among the Third Element employing a battery of silver carbonate impregnation methods. Modern technology reconfirmed del Río Hortega’s discoveries, and demonstrated that altered forms of microglia participate in the pathology of AD, since they are a key component of senile plaques (64–67) and other lesions (67). To the best of our knowledge, it has not been proposed before that schizophrenia results from targeting the 4EC, yet there seems to be no reason precluding such a possibility, which, of course, needs to be assessed scientifically. Part of the reasons to pursue such an assessment is precisely to establish yet another parameter for comparison with AD, unencumbered by the titillating possibility that 4E targeting in schizophrenia might be the long-sought but elusive “cause” of the disorder. The latter will obviously necessitate experimental evaluation, which is barely underway.

## Caveats on a Cellular-Level Formulation

Given the ongoing dramatic changes in the distinction between neurons and glial cells in the brain, the above proposal that 4EC are pivotally involved in AD and schizophrenia defies the former notion that the targeted element(s) can be conceptualized simplistically as neurons vs. macroglia vs. microglia, vs. other cells that are not of a neural lineage. In fact, considering the still unresolved nature of 4EC as precursors or variants of oligodendrocytes, their near-neuronal attributes (e.g., synapses and the participation on glutamatergic and GABAergic neurotransmission) and their features in common with “conventional” astrocytes, their postulated primary, even pathogenic, involvement in AD blurs the previously unassailable distinction among all conventional cell types in AD. Even if the present proposal about the predominant targeting of 4EC in AD does not survive experimental testing, it casts doubt on the ability to postulate targeting mechanisms based exclusively on neuronal vs. glial phenotypes. Thus, it is also possible that it is not specific cell types, but key attributes shared between neuronal and non-neuronal cells that are targeted early on in the disease process, if this process attacks first – or in any way selectively – neural cells. Similar considerations apply to schizophrenia, in which, as stated in the preceding section, the characterization of the possible participation of 4EC has barely begun solely by stating the possibility of such a mechanism.

## The Innovation Gap and Translational Applications to the Prevention and Treatment of AD and Schizophrenia

Progress in conquering AD, schizophrenia, and related disorders is hampered by the so-called Innovation Gap (IG), which is a collection of challenges that includes: (a) the fact that the number of approved treatments has not increased despite substantial increments in the investment toward the discovery and testing for this purpose over decades, (b) the frequent lack of predictive power for the efficacy in humans of the outcomes of testing in so-called “models” of the disorder, and (c) in the case of AD, the failure of all trials published to date based on the overwhelmingly dominant amyloid hypothesis of AD pathogenesis (20, 21). If the 4EC hypothesis is correct, it would open an essentially unexplored avenue for research and treatment development that could have a powerful effect in overcoming the IG. Similar challenges apply to schizophrenia, given the shared lack of an understood etiology and pathophysiology, and despite the larger number of therapeutic agents available since these – like in AD – palliate but do not “cure” the disorder.

A recent report provides powerful evidence that it may now possible to predict the outcome of patients at risk of developing AD, by measuring putative markers of the disorder that include β-amyloid and both total and phosphorylated tau protein in the cerebrospinal fluid (68). However, markers with proven diagnostic value do not presently include indicators of inflammation, macroglial alterations, and, much less, 4EC involvement, although there are numerous reports suggesting – but not proving – that the former may also be of diagnostic value (69–73). Given the probable multifactorial nature of sADs (54), it is likely that an array of markers addressing different mechanisms involved in the condition may help to: (1) increase the sensitivity and specificity of the tests under development, (2) discern subtypes of patients in which different mechanisms mediate a similar cognitive impairment syndrome, and (3) predict and monitor the response to various alternative mechanism-selective treatments. Therefore, whether the hypothetical 4EC mechanism proposed here is universal, or unique to certain groups of patients, research aimed at testing this hypothesis may lead to the implementation of assays for 4EC involvement by cerebrospinal fluid analysis, or blood-borne indicators of 4EC involvement, or *in vivo* imaging assessment of such involvement (with ligands for 4EC) – or a combination of these – as well as a more refined histopathological assessment for patients with Mild Cognitive Impairment and dementia *postmortem*. The above considerations are relevant also to our recent assessment of the intersection of the epidemics of dementia and diabetes, since this is one additional situation in which the elucidation of the targeted vs. spared or disease-resistant elements is essential to move beyond conflicting interpretations of the same or closely related experimental observations (74). Very similar considerations apply to schizophrenia, given the equivalent lack of biomarkers both to make the diagnosis unencumbered by clinical observations, as well as to measure the efficacy of the treatments and to determine whether they impact the long-term prognosis of individual patients (45, 75). This dilemma includes, but is not limited to the high prevalence of metabolic syndrome and diabetes associated with the use of antidepressants and antipsychotics in schizophrenia (76), which, like in AD (74), probably interact with the original mechanisms responsible for brain dysfunction and is quite likely to influence the well-documented tendency for patients with schizophrenia to continue to deteriorate cognitively, and certainly prematurely, even now that their long-term survival has improved with better treatment (45).

It has not escaped our attention that casting hypotheses on the primary targeting mechanisms causing AD and schizophrenia in terms of one or another cell type – or cellular component, such as synapses – may be narrow-minded and ultimately inadequate. In fact, given: (1) the seemingly “unconventional” nature of 4EC, which share cytological and molecular features with neurons, macroglia, and oligodendrocytes, but are neither, (2) the evidence that markers previously felt to be unique to neurons – such as doublecortin – or macroglia – such as S100B – are expressed by a variety of other cell types (77, 78), and (3) that the S100B/RAGE-mediated activation of microglia (79) indicates there may be and inextricable interaction between 4EC, macro- and microglia in the genesis of brain inflammation that initially triggers neurodegeneration (54, 80, 81), it may be difficult to discern between a primary/initial macro- vs. microglial trigger of AD or schizophrenia. This challenge is in fact compounded by the 4EC hypothesis, since 4EC share properties with several neural cell types. However, it would seem that casting such a hypothesis in terms of a putative primary involvement of 4EC is scientifically testable, and provides a heuristically useful counterbalance to the presently massive – if appropriate – attention to the suspected role of microglia in the early mechanisms mediating AD and schizophrenia (54, 66, 67, 82). This will require further characterization of 4EC in both the normal and diseased human and non-human brain, to approach the level of sophistication with which it is possible to study today the involvement of more “conventional” types of cell in the brain and other organs. None of the above hypothetical scenarios are contradictory or incompatible with the eventual (i.e., secondary) dysfunction, followed by the degeneration and death of neurons, as the final mediator of the clinical manifestations of AD, schizophrenia, and related disorders as proposed by many authors, including ourselves (19–21, 54, 74, 83). Such secondary events include also inflammation and the (intra- and extra-cellular) accumulation of pathological forms of amyloid and tau proteins (in AD, since it is unknown whether this occurs in schizophrenia), regardless of whether these are cast as disease-compounding, or ultimately ineffective or counterproductive attempts at cellular and molecular defense.

## Final Considerations

It is important to remark that future research in both schizophrenia and AD is likely to benefit from substantial refinements in non-pharmacological treatments available today, which are unfortunately poorly standardized at present, and, therefore, not regimented into accepted treatments covered by virtually all forms of health insurance. Perhaps the best example of such a need is physical exercise, for which there is a growing body of literature indicating clearcut benefits not only in mild cognitive impairment and AD (84), but also in schizophrenia (85). The significant elevation of brain-derived neurotrophic factor (BDNF) caused by physical exercise – which has also been used as a surrogate marker for “neuroplasticity” – is among the likely mediators of such experimentally assessed benefits, which have been corroborated repeatedly and independently, along with as yet much less well standardized attempts at cognitive remediation (a.k.a. “cognitive retraining”) and various forms of social, sensory, and motor stimulation. These interventions appear to synergistically improve the condition of patients with both conditions (AD and schizophrenia), in addition to pharmacological agents. Therefore, while never ignoring or neglecting the need to continue searching for the hypothetical “cause(s)” of these disorders, and refining our intellectual and therapeutic constructs for them as clinical syndromes vs. true diseases, we should continue to develop interventions such as exercise, various forms of sensory stimulation, and cognitive retraining with the intention that they become a mainstay of treatment alongside more “conventional” (e.g., pharmacological) approaches. This is indispensable, since these novel forms of intervention appear to be strongly beneficial, and perhaps overall much less expensive and devoid of severe side effects than seemingly more “conventional” approaches to molecular therapy that, in any case, remain only palliative and unsatisfactory in the eyes of virtually all practitioners. This approach should ideally be part and parcel of the ongoing re-examination of the field of neurodegenerative and neuropsychiatric disorders, which has as a common theme the dire need to reinvigorate the intellectual framework to establish causality, etiologies, and pathophysiology in an integrative, multidisciplinary fashion, beyond the molecular level alone (20, 21, 86). Taken together, these considerations emphasize the need to continuously – and increasingly – “compare notes” among specialists in AD and schizophrenia, as well as many other closely related fields. This exchange will likely continue to result in intellectual “cross-pollination” that will facilitate our cruising the arduous road to conquer these and many other related neuropsychiatric disorders.

## Conflict of Interest Statement

The authors declare that the research was conducted in the absence of any commercial or financial relationships that could be construed as a potential conflict of interest.
